# Using Rhodamine to Tag Mites for Studies of Pre‐ and Post‐Copulatory Sexual Selection

**DOI:** 10.1002/ece3.70525

**Published:** 2024-11-05

**Authors:** Anastasia J. Shavrova, Bruno A. Buzatto, Michael M. Kasumovic

**Affiliations:** ^1^ Evolution and Ecology Research Centre, School of Biological, Earth and Environmental Sciences UNSW Sydney Sydney New South Wales Australia; ^2^ College of Science and Engineering, Flinders University Bedford Park South Australia Australia

**Keywords:** animal behaviour, invertebrates, sexual selection, tagging, tracking

## Abstract

Our understanding of sexual selection is advancing with new technologies that tag individuals or their sperm, revealing how females use post‐copulatory processes to discriminate between competing mates. Many tagging methods have been devised primarily for model insect organisms like *Drosophila* or Gryllidae. Developing such novel methods, however, is expensive and requires intensive investment. In this experiment, we trial the use of Rhodamine B (RhB) and Rhodamine 110 (Rh110) in a small arachnid, the bulb mite *Rhizoglyphus echinopus*, for pre‐ and post‐copulatory observations as it is a relatively inexpensive and simple way to tag individuals and their ejaculate proteins. First, we tested whether RhB and Rh110 applied to food can be used as a tagging method to track and distinguish between individuals. Second, we explored whether Rhodamine applied in this way can be used to track sperm transfer. We found that both tagging probes worked well in tagging individuals and that we were able to distinguish between individuals using both LED and fluorescent microscopy. We also found that Rhodamine degraded rapidly in the animals, likely due to their fast metabolism. Due to the rapid degradation, we observed variable results in the sperm transfer trials. We suggest multiple uses for Rhodamine and highlight other invertebrates where this method may come into use for the study of sexual selection.

## Graphical abstract

We trial the use of Rhodamine, for the first time in arachnids. We first trial the method for behavioural ecology and evolution trials to track the individuals within large population sizes. Furthermore, we trial this method for post‐copulatory studies that investigate sperm competition and female cryptic choice.

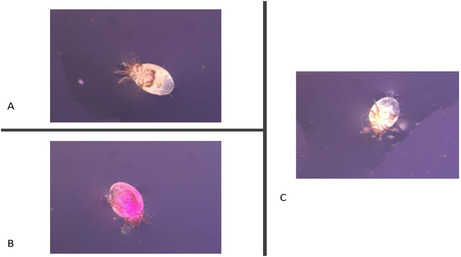

## 1 Introduction

Sexual selection results from differential access to gametes for fertilisation (Shuker and Kvarnemo 2021) and can be studied from multiple different angles. To truly understand how sexual selection functions, it is crucial to estimate its strength before and after mating, especially as these processes can interact and influence overall reproductive success (Lande and Arnold 1983). Pre‐copulatory sexual selection refers to the competition or mate‐choice processes occurring before mating, whereas post‐copulatory sexual selection encompasses processes that happen after mating has occurred, such as sperm competition and cryptic female choice (Jones and Ratterman 2009). Pre‐ and post‐copulatory behaviours and the associated fitness of individuals expressing them are often used to infer the strength of sexual selection on sexual traits.

Most researchers estimate the strength of selection from pre‐copulatory behaviours, as it only requires the observation of natural behaviours, such as the ability of an individual to secure and defend a territory or resource (Dubois and Giraldeau 2005; Grant 1993), the ability to outcompete a rival in direct competition (Parker 1974), or the number of matings and order in which individuals mate (Jordan, Kokko, and Kasumovic 2014). However, while observing behaviours in natural or laboratory habitats, we are often limited to a maximum number of individuals we can track simultaneously. Successfully observing and identifying individuals across a wide variety of species and in more representative social conditions is essential if we want to extrapolate these results to natural behaviours and situations across the animal kingdom.

Despite the ubiquitous use of behavioural observations to estimate the strength of selection before copulation, our ability to identify and track individuals under more natural circumstances in the field and in the laboratory can be difficult. For example, experiments aimed at identifying the traits that lead to a successful mating are often limited to pairs of interacting males and females (Wagner 1998). If multiple males are used, individuals must be marked to distinguish between them (Jung et al. 2020). Although this is relatively simple in vertebrates, it is more difficult in invertebrates as they are smaller, have fewer identifying features and often occur in large numbers. There are different tagging techniques developed for use in invertebrates (particularly insects), such as externally marking with fluorescent dusts, paint, ink and body mutilation (see review Hagler and Jackson 2001). These methods are usually inexpensive and relatively easy to apply, however can be toxic to the animal if applied incorrectly (Hagler and Jackson 2001), can alter the animals' behaviour (Still et al. 2014), and some of the techniques are not permanent, meaning that individuals cannot be tracked for long periods (e.g., fluorescent dusts). A further problem is that if individuals are small and occur in large numbers, such as fruit flies (Grimaldi and Jaenike 1984) or mites (Radwan 1995), inexpensive and easy marking techniques can become expensive and complex to perform accurately for too many individuals.

Post‐copulatory estimations of sexual selection include measuring the number of offspring an individual produces, which is difficult when females mate with multiple partners, as it requires identifying the sires of the offspring through genetic markers (McClure et al. 2012) or sterile male techniques (Scott and Williams 1994). For example, the green fluorescent protein (GFP) in *Drosophila melanogaster* (Manier et al. 2010) is often used to make post‐copulatory observations. However, the costs of developing such techniques are high and may not be amenable to small behavioural projects. Additionally, the observation of fluorescence can only be done under an expensive confocal microscope with fluorescent functions (Manier et al. 2010; Remington 2011) and may have effects on protein function (Michaelson and Philips 2006), which can affect sperm count and quality. Furthermore, visible genetic markers such as eye colour or colour morph used in mating experiments are often limited to two males because of the recessive and dominant relationship of the genes involved (Balfour et al. 2018; Filice and Dukas 2019).

Studying sexual selection in colony‐dwelling animals can bring additional challenges. Colony‐dwelling animals also often exhibit unique reproductive strategies, such as intense sperm competition (Simmons 2005; Smith 2012), which can also play into conflict or cooperation between or within sexes. Some species of the class Arachnida are colony‐dwelling animals, making them difficult to study under more natural circumstances. Arachnids are particularly interesting to study in regard to sexual selection and conflict because of their extreme sexual cannibalism (Schneider 2014) and morphological dimorphism (McLean, Garwood, and Brassey 2018). However, sexual behaviours are difficult to study in arachnids for several reasons. First, the prevalence of multiple mating opportunities makes it difficult to assess individual reproductive success of males (Smith 2012). Second, the unique and variable genitalia and sperm of arachnids render most post‐copulatory processes cryptic (Eberhard and Huber 2010). Lastly, large population numbers can obscure individual behaviours and interactions, posing challenges for detailed individual observations (Radwan 1995). Most pre‐copulatory behaviours of arachnids are usually observed in unnatural conditions where mating is limited to one or, at most, two mating partners. Individuals are often identified with acrylic paint (e.g., Rypstra 1985) and fluorescent dust (e.g., Still et al. 2014), both of which can have practicality issues (Evans and Gleeson 1998) and can affect an animal's behaviour (Still et al. 2014). Additionally, post‐copulatory observations are often limited by the extended generation time of some arachnids (Murrell et al. 2005; Schmoller 1970) or by female cryptic choice (Eberhard 1997); therefore, transgenic methods are not practical. This is why male sterilisation through radiation is commonly used (Christenson et al. 1986), although this technique can alter feeding behaviour, reaction to light, decrease locomotion, chemoreceptivity (Langley, Curtis, and Brady 1974), mating vigour and success, as well as the competitiveness of individuals (Oliva et al. 2012).

In this study, we trial Rhodamine for use in tracking individual bulb mites (*Rhizoglyphus echinopus*), a colony‐dwelling arachnid. Rhodamine is a fluorescent probe that binds to proteins in the animal, including the ejaculate, which can be used as a marker for mating studies. Rhodamine has been used in invertebrates such as leafhoppers (Hayashi and Kamimura 2002), fireflies (van Reijden, Monchamp, and Lewis 1997) and moths (Blanco et al. 2006; Sparks and Cheatham 1973), to observe male ejaculate and spermatophores in a female's reproductive tract. Rhodamine is a cost‐effective way to stain sperm, oocytes or individuals and can be observed by the naked eye, under a LED of a microscope, a fluorescent laser (Blanco et al. 2006; Hayashi and Kamimura 2002; Sparks and Cheatham 1973). Using Rhodamine is also time‐efficient, as it does not require back‐crossing individuals into a population as in a GFP approach (Manier et al. 2010) and can instead be injected into larger invertebrate animals like moths (Sparks and Cheatham 1973), spermatophore (van Reijden, Monchamp, and Lewis 1997), female reproductive tract (Hayashi and Kamimura 2002) or fed to the animal by mixing the dye into their diet (Blanco et al. 2006; Sparks and Cheatham 1973). Rhodamine has been shown to have little to no effect on the lifespan (Blanco et al. 2006) or mating behaviour (van Reijden, Monchamp, and Lewis 1997) of animals, although such studies with Rhodamine have been done in insects, and its potential to be used in arachnids remains unknown.

*R. echinopus* males and females are likely polygynandrous (Radwan 2009), live in colonies, and the males are polyphenic (Radwan 1995, 2001). Polyphenisms refer to an extreme case of phenotypic plasticity where one gene can express multiple discrete phenotypes triggered by an environmental cue (Yang and Pospisilik 2019). In the case of *R. echinopus*, the polyphenism is triggered by colony density (Radwan 2001) and juvenile size. When density is high, most males will moult into the scrambler male morph and use a passive sneaker tactic. When the density is low, males will moult into a fighter male morph and use a weaponised, mate‐monopolising tactic (Radwan 2001, 2009). Because individuals are so small, it is impossible to use traditional marking techniques used in other invertebrates (Hagler and Jackson 2001). This means that mating trials are usually limited to observing single pairs, which can give an inaccurate representation of mating preference, fitness and intrasexual competition (Anderson, Kim, and Gowaty 2007; Shackleton, Jennions, and Hunt 2005). This limits our ability to explore individual behaviours and how the strength of selection may vary because of shifts in the relative density of morphs. Rhodamine, however, offers the opportunity to tag individuals by staining the food (yeast) that they eat. Our aims were thus to (1) test Rhodamine B (RhB) and Rhodamine 110 (Rh110) as a tagging method by observing any colour change to the individuals or by the presence of fluorescence under a microscope to track and identify individuals and to (2) determine whether Rhodamine could be used to track sperm transfer by observing he presence of fluorescence in the female or her eggs.

## 2 Methods

### 2.1 Methods Overview

To achieve our first aim of testing RhB and Rh110 as effective tagging methods for behavioural observations, we conducted several tests. First, we tested different concentrations of Rhodamine solutions fed to male *Rhizoglyphus echinopus* and compared various mounting media to determine any visual differences between mites with and without Rhodamine through LED and fluorescent microscopy observations. Furthermore, we assessed the degeneration of Rhodamine to better understand how long the fluorescence could be reliably observed.

For our second aim of testing RhB and Rh110 as effective methods to observe sperm transfer, we mated males tagged with Rhodamine and observed the presence or absence of fluorescence in the females and their eggs.

### 2.2 Rhodamine Description

Rhodamine is a fluorescent probe used for the fluorescent labelling of proteins. The fluorescence is created by the presence of a planar, multi‐ring aromatic xanthene core structure with nitrogen in place of oxygen atoms in the outer rings (Beija, Afonso, and Martinho 2009; Hermanson 2008). Rhodamine B contains two ethyl groups on each nitrogen and a carboxylate group at the 3^rd^ position of its lower ring, whereas Rh110 contains no substituents on the upper nitrogens and the carboxylate on the lower ring. RhB has an excitation wavelength of 546 nm and emission wavelength of 568 nm, whereas Rh110 has an excitation wavelength of 500 nm and emission wavelength of 522 nm. Both reagents are water soluble (Hermanson 2008).

### 2.3 *Rhizoglyphus echinopus* Stock Population

The stock populations of *Rhizoglyphus echinopus* used in this study are descendants of a population sourced off an infested organic onion purchased in August 2005 from a health food shop in Perth, WA (Buzatto, Simmons, and Tomkins 2012). The descendants of these populations were subsequently maintained at UNSW Sydney in New South Wales from 2019. We housed the mites in six 90 mm Petri dishes partially filled with Plaster of Paris, which were kept inside closed food containers. We placed the containers in dark incubators at a temperature of 22°C. Distilled water was regularly sprayed to maintain > 90% humidity level. The mites were provided with Allinson's dried yeast as a food source and tissue paper as a substrate, ad libitum. To preserve genetic diversity within the cultures, a small proportion of mites were periodically transferred between Petri dishes. All 300 individuals used to test the protocol were virgins sourced from 20 females subsetted from the stock population. We isolated on average 25 larvae from these females and reared them individually in small cylindrical glass vials (diameter = 100 mm and height = 14 mm; hereafter referred to as vial) with a Plaster of Paris base (4–5 mm thick) on top of damp filter paper in a 90 mm Petri dish in a food container. We closed the vials with a small piece of cotton wool.

### 2.4 Rhodamine Set‐Up

#### 2.4.1 Concentration

We tested two solution concentrations for our protocol. We mixed 4.17 mM of RhB and Rh110, as used in van Reijden, Monchamp, and Lewis (1997), referred to as the original concentration, and we doubled this concentration to 8.34 mM of Rh100 and RhB, referred to as the doubled concentration. We mixed the solution with 2 mg of yeast, shaking the mixture well until it was homogenous. We pipetted 0.25 mL of each food solution onto the Plaster of Paris substrate of a vial with one individual mite—henceforth these vials are referred to as Rhodamine vials. We only fed the Rhodamine solution to male bulb mites, as female bulb mites would be the recipients of the Rhodamine‐stained ejaculate. In total, 108 males (approximately five males from each parental female) were fed the Rhodamine solution for 24 h minimum before each testing protocol (RhB original concentration *n* = 27 males, RhB double concentration *n* = 27 males, Rh110 original concentration *n* = 27 males, Rh110 double concentration *n* = 27 males). While some methods inject Rhodamine into the animal (e.g., Sparks and Cheatham 1973), we are unable to do this with *R. echinopus*, as they are too small and fragile.

#### 2.4.2 Mounting Media

We tested four different mounting media for our protocol: distilled water, Fluoromount, Immu‐mount and phosphate‐buffered solution (PBS). We used the distilled water medium for the light‐emitting diode (LED) illumination trials only, whereas we used Fluoromount, Immu‐mount and PBS for the fluorescent illumination trials. Before we placed an individual male into the mounting medium, we washed them in a droplet of distilled water on a Petri dish to remove any excess Rhodamine solution stuck to its body. We mounted all individuals for the fluorescent trials ventral side up on a 76.2 × 25.4 mm microscope slide with the cover slip placed gently on top to avoid squishing the mites. We mounted three males in PBS for each Rhodamine treatment and concentration (total *n* = 12), and 12 males in either Fluoromount or Immu‐mount for each Rhodamine treatment and concentration (total *n* = 96). We cured the slide for a minimum of an hour in a closed container to minimise light exposure. Alongside the Rhodamine male mites, we always mounted a negative control male, which consisted of a male that was not fed Rhodamine, for RhB (*n* = 3) and Rh110 (*n* = 3). The males died during the mounting process likely due to dehydration caused by the curing of the mounting liquid. The three negative control males were re‐used as no treatment was used on them, and thus, their fluorescence should not have changed between microscope viewings.

#### 2.4.3 LED Illumination

To visually distinguish between individuals, we cleaned mites in a droplet of distilled water and visually observed the presence of a yellow (Rh110) or purple (RhB) hue in the body of 10 male mites from each Rhodamine treatment and concentration (total *n* = 40) under an Optico ASZ‐200 Stereo Microscope. The mites were alive for all the LED illumination observations.

#### 2.4.4 Fluorescent Illumination

To observe the fluorescence of the Rhodamine‐fed male mites, we used a Zeiss LSM 780 or Zeiss LSM 880 microscope with the 10× (0.45 DICII) objective. To assess RhB fluorescence, we used a laser with 514 wavelength and the range indicator from 525 to 740 nm. To assess Rh110 fluorescence, we used a laser with 488 wavelength and the range indicator from 499 to 696 nm. We always started with a positive control sample—a male mite that had been fed the highest concentration of Rhodamine and/or fed the solution most recently—and would therefore fluoresce the brightest. We set the laser gain so the fluorescence was visible but not oversaturated. We then compared all subsequent males to the positive control laser gain. Negative controls were used to confirm the absence of fluorescence in the specimen and reveal any background fluorescence by using samples that had not been exposed to Rhodamine, ensuring that any fluorescence observed in the test samples was due to the fluorescent probe. We took two images of each sample, the laser image with fluorescence and a transmission‐photo multiplier (TMP) image as a reference image. The image was always focused on the genitalia of the mite.

#### 2.4.5 Degeneration

We tested whether Rhodamine degenerates in the male mite, and if so, how quickly. After males were fed their Rhodamine solution for at least 24 h, we washed the male in a droplet of water and placed him into one of three treatments: a vial with the Rhodamine yeast solution (*n* = 36 RhB males, *n* = 36 Rh110 males), a vial with yeast containing no Rhodamine (*n* = 33 RhB males, *n* = 33 Rh110 males), an empty vial without yeast (*n* = 36 RhB males, *n* = 36 Rh110 males) and a negative control (*n* = 3 RhB males, *n* = 3 Rh110 males). After 24 h in these vials, we mounted half of the males of each treatment in Fluoromount and Immu‐mount, ventral side up. For this examination, we no longer used the PBS mount after examining the mounting medium results. After at least 1 hour of curing the slides in a closed container, we examined the slides under the microscope Zeiss LSM, where all males were compared with the positive control. We euthanised the mites during the mounting process for this observation.

### 2.5 Mating Protocol

To determine whether the Rhodamine is binding to the ejaculate of male mites and whether it is transferred to females, we fed virgin males a Rhodamine solution by pipetting it onto the Plaster of Paris in the vial and allowing the mites feed on the solution ad libidum for a minimum of 24 h. We then divided those individuals into five treatments: (1) virgin males fed and left in their Rhodamine vial (positive control; *n* = 32 RhB males, *n* = 32 Rh110 males), (2) virgin males fed Rhodamine first then moved from their Rhodamine vial and placed in an empty vial (degeneration control; *n* = 54 RhB males, *n* = 54 Rh110 males), (3) virgin males fed Rhodamine and then mated with females in an empty vial (male treatment; *n* = 46 RhB males, *n* = 46 Rh110 males), and (4) a negative control of virgin males not fed Rhodamine (*n* = 3 RhB males, *n* = 3 Rh110 males). We also considered the females mated with Rhodamine‐treated males as an additional treatment (5) to observe the presence or absence of Rhodamine fluorescence after mating, as this would have confirmed sperm transfer from a Rhodamine‐fed male (female treatment; *n* = 33 RhB females, *n* = 33 Rh110 females). We did not have further negative controls of non‐Rhodamine‐tagged males or females, as the negative controls are solely used to mark the absence or presence of background fluorescence, and the mating status of the individual would be unlikely to change this. We made sure the males and females mated by checking the vials every 10 min until a male mounted a female. Mating would take approximately 2 hours, and therefore, we kept the virgin males in the positive control and degeneration control in their vials for the same amount of time as the mating treatment. After the pair was finished mating, we mounted all individuals from the five treatments ventral side up in Immu‐mount, euthanising them in the process. We observed the individuals by examining the positive control first and comparing all subsequent samples to the positive control using the same laser gain settings.

To determine whether the Rhodamine from male ejaculates was integrated into the eggs, we isolated four females in total (two females mated with an RhB‐fed male and two males mated with an Rh110‐fed male) and collected their eggs 3 days after mating. This treatment followed our initial mating protocol. Given that we observed very little fluorescence in the mated females, we initially trialled only four females to assess the presence of any fluorescence in the eggs before considering an increase in the sample size for greater robustness. However, we used a negative control of eggs that came from females that had never encountered Rhodamine‐fed males. We mounted a subset of the eggs in Immu‐mount and observed them under the microscope. The females were not euthanised during the egg collection process, but it is likely that the offspring in the eggs died during the mounting process. There were Rhodamine yeast particles on the slide which we used as a positive control.

### 2.6 Analysis

We used Fiji, an extended version of the biological image analysis program ImageJ (Schindelin et al. 2012), to measure the specimens' fluorescence for concentration, mounting medium, degeneration and mating protocols. For each laser image, we took three replicate measurements with the circle tool: area of the selection, integrated density of the selection in the image of the mites' body near the genitalia and three replicate measurements of the background (see Figure S1 for reference). The TMP image of the mite was used for reference when measuring the fluorescence (or lack thereof) near a sample's genitalia. We then calculated the corrected total cell fluorescence (CTCF) of each sample through the formula:

$$\begin{array}{c} \begin{array}{c} \text{𝐶𝑇𝐶𝐹}=\text{𝐼𝑛𝑡𝑒𝑔𝑟𝑎𝑡𝑒𝑑 𝐷𝑒𝑛𝑠𝑖𝑡𝑦}-\text{(𝐴𝑟𝑒𝑎 𝑜𝑓 𝑠𝑒𝑙𝑒𝑐𝑡𝑖𝑜𝑛}\\ \times \;\text{𝑓𝑙𝑜𝑢𝑟𝑒𝑠𝑐𝑒𝑛𝑐𝑒 𝑜𝑓 𝑏𝑎𝑐𝑘𝑔𝑟𝑜𝑢𝑛𝑑)} \end{array} \end{array}$$

To test for CTCF differences due to the solution concentration or the mounting medium, we used a linear model including the concentration, mounting medium and their interaction as fixed effects. To test for CTCF degeneration, we used a linear model with treatment, mounting medium and their interaction as fixed effects. To test for CTCF variations we ran a linear model with mating status as a fixed effect. Mating status was defined with the following groups: virgin control (virgin male not fed Rhodamine), positive control (Rhodamine‐fed virgin male left in the vial with Rhodamine), Rhodamine‐fed virgin male moved to an empty vial for 24 h, Rhodamine‐fed mated male and mated females (mated to a Rhodamine‐fed male). All models had a Gaussian error distribution. We used R version 4.1.3 (R Core Team 2022) for all our analyses. We obtained all estimated marginal means, and Tukey contrasts with the ‘emmeans’ package (Lenth 2022).

## 3 Results

All our *Rhizoglyphus echinopus* mites survived the 24 h in the feeding Rhodamine treatments.

### 3.1 Individual Tagging

#### 3.1.1 LED Illumination

**FIGURE 1:**
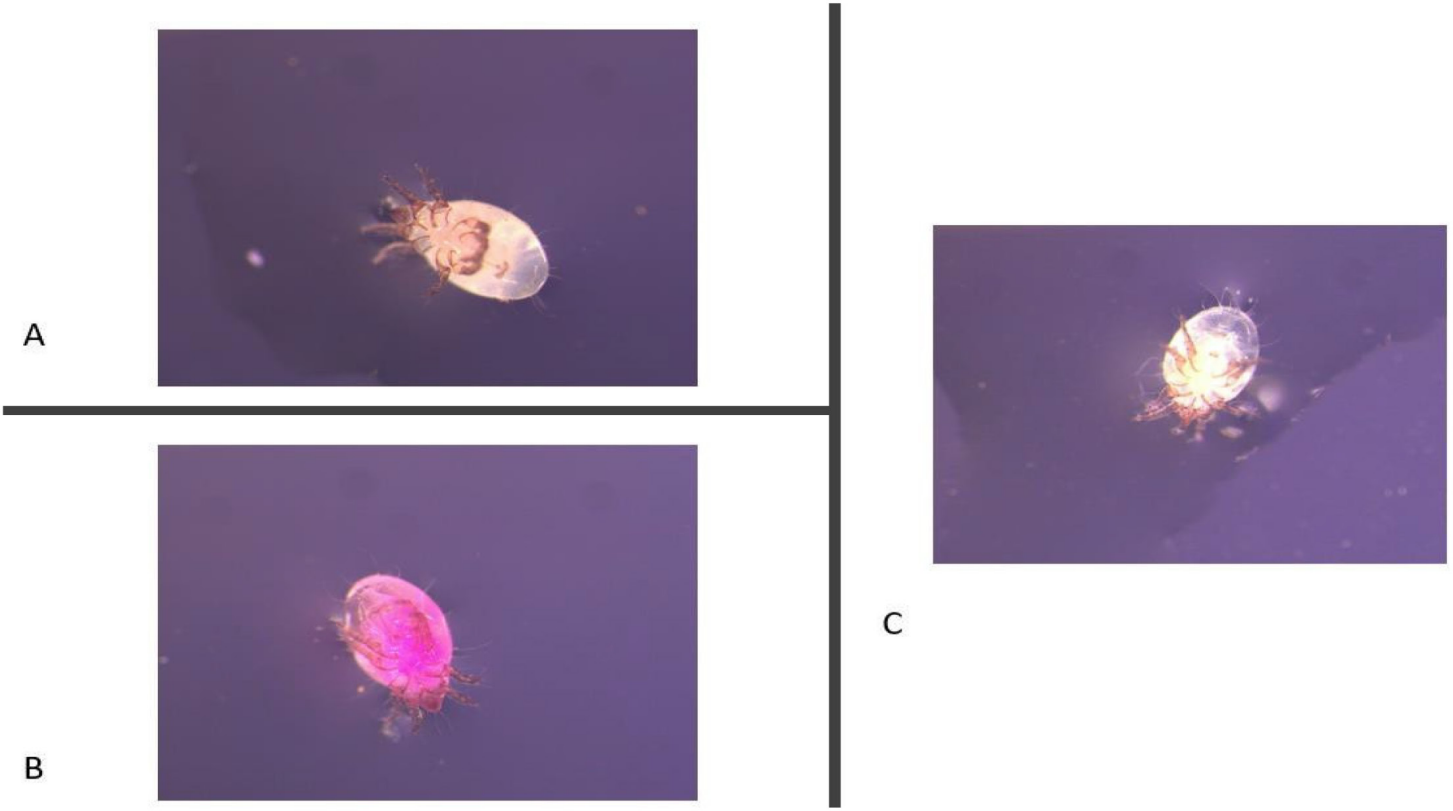
Male mites fed (A) Rh110 solution with yeast for 24 h, (B) RhB solution with yeast for 24 h and (C) yeast with no Rhodamine.

We were able to visually assess male mites fed RhB and Rh110 under the stereo microscope. Males fed Rh110 (Figure 1A) had a dark orange tint and males fed RhB (Figure 1B) had a purple tint inside their bodies when compared to unmarked mites (Figure 1C).

#### 3.1.2 Fluorescence Illumination

**FIGURE 2:**
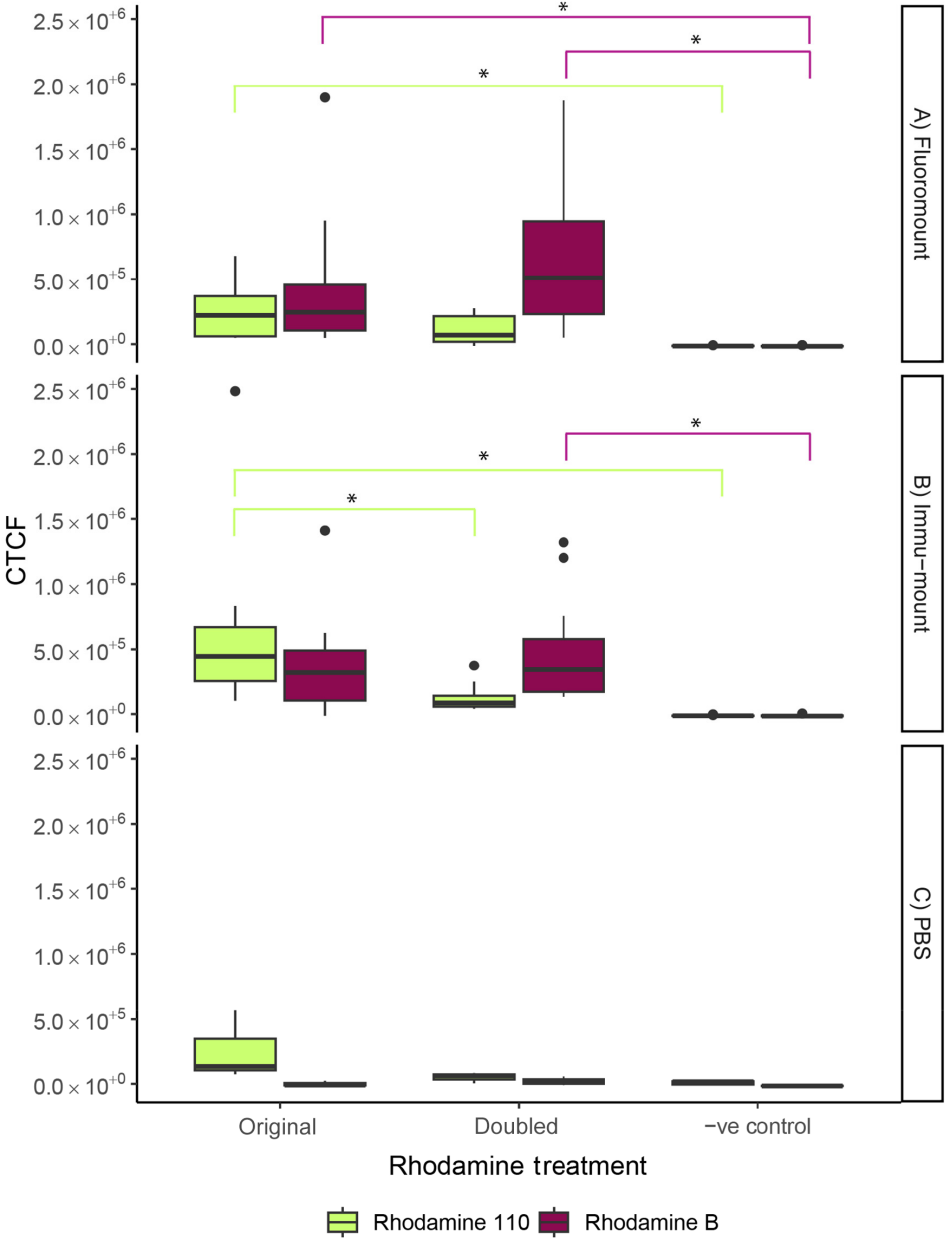
Corrected total cell fluorescence (CTCF) of the Rhodamine treatment for original (4.17 mM) and doubled (8.34 mM) concentrations with the negative control in mounting medium (A) Fluoromount, (B) Immu‐mount and (C) PBS. Significant differences are outlined using an asterisk and lines matching the colours of the treatment.

RhB fluorescence did not differ significantly between the original or doubled concentration within the same mounting medium (Figure 2; Table S1). We did, however, see a difference between the negative control and the concentrations within the same mounting medium. Both the original and doubled concentration of RhB fluoresced significantly more than the negative control in Fluoromount (Figure 2A; Table S1). In contrast, only the doubled concentration fluoresced more than the negative control in Immu‐mount (Figure 2B; Table S1). No treatment differed in fluorescence from the negative control in PBS (Figure 2C; Table S1; See Figure S2 for images of fluorescence).

Rh110 fluorescence differed significantly between the original and doubled concentration in Immu‐mount (Figure 2B; Table S1). Only the original concentration of Rh110 fluoresced significantly more than the negative control in both Fluoromount (Figure 2A; Table S1) and Immu‐mount (Figure 2B; Table S1). In PBS, no treatment differed in fluorescence from the negative control (Figure 2C; Table S1; See Figure S2 for images of fluorescence).

### 3.2 Degradation of Fluorescence

**FIGURE 3:**
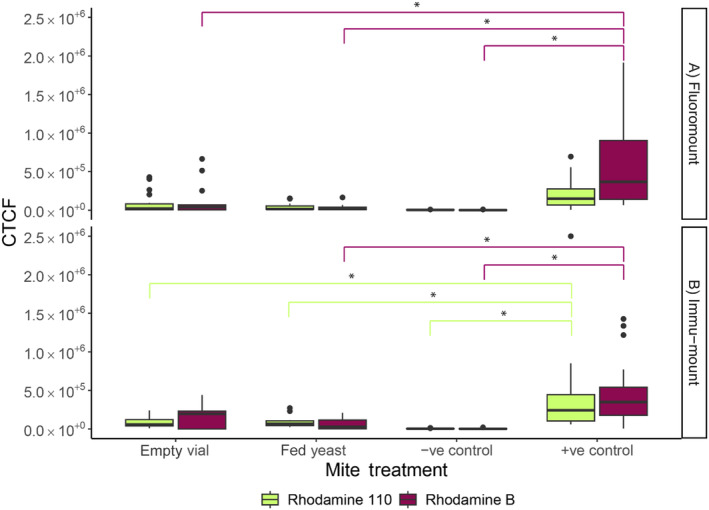
Corrected total cell fluorescence (CTCF) of the Rhodamine degradation treatment for males kept in empty vials for 24 h after treatment, fed yeast for 24 h after treatment, negative control male that was never fed Rhodamine, and positive control male that was kept in a Rhodamine vial for 24 h, in mounting medium (A) Fluoromount and (B) Immu‐mount. Significant differences are outlined using an asterisk and lines matching the colours of the treatment.

Next, we explored how samples degraded once the male mite was fed the solution. RhB‐fed males that were either fed yeast or put into empty vials for 24 h fluoresced similarly to the negative control and significantly less than the positive control in Fluoromount (Figure 3A; Table S2). In Immu‐mount, the males that were left in an empty vial for 24 h fluoresced similarly to the positive control (Figure 3B; Table S2), whereas the males that were fed yeast fluoresced significantly less than the positive control with no difference from the negative control (Figure 3B; Table S2). The negative control fluoresced significantly less than the positive control in both Immu‐mount and Flouromount (Figure 3; Table S2).

Rh110‐fed males that were either fed yeast or put into empty vials for 24 h did not fluoresce differently from the negative control in both Fluoromount and Immu‐mount and were significantly lower in fluorescence than the positive control in Immu‐mount (Figure 3; Table S2). The negative control fluoresced significantly less than the positive control only in Immu‐mount (Figure 3; Table S2).

### 3.3 Sperm Transfer

**FIGURE 4:**
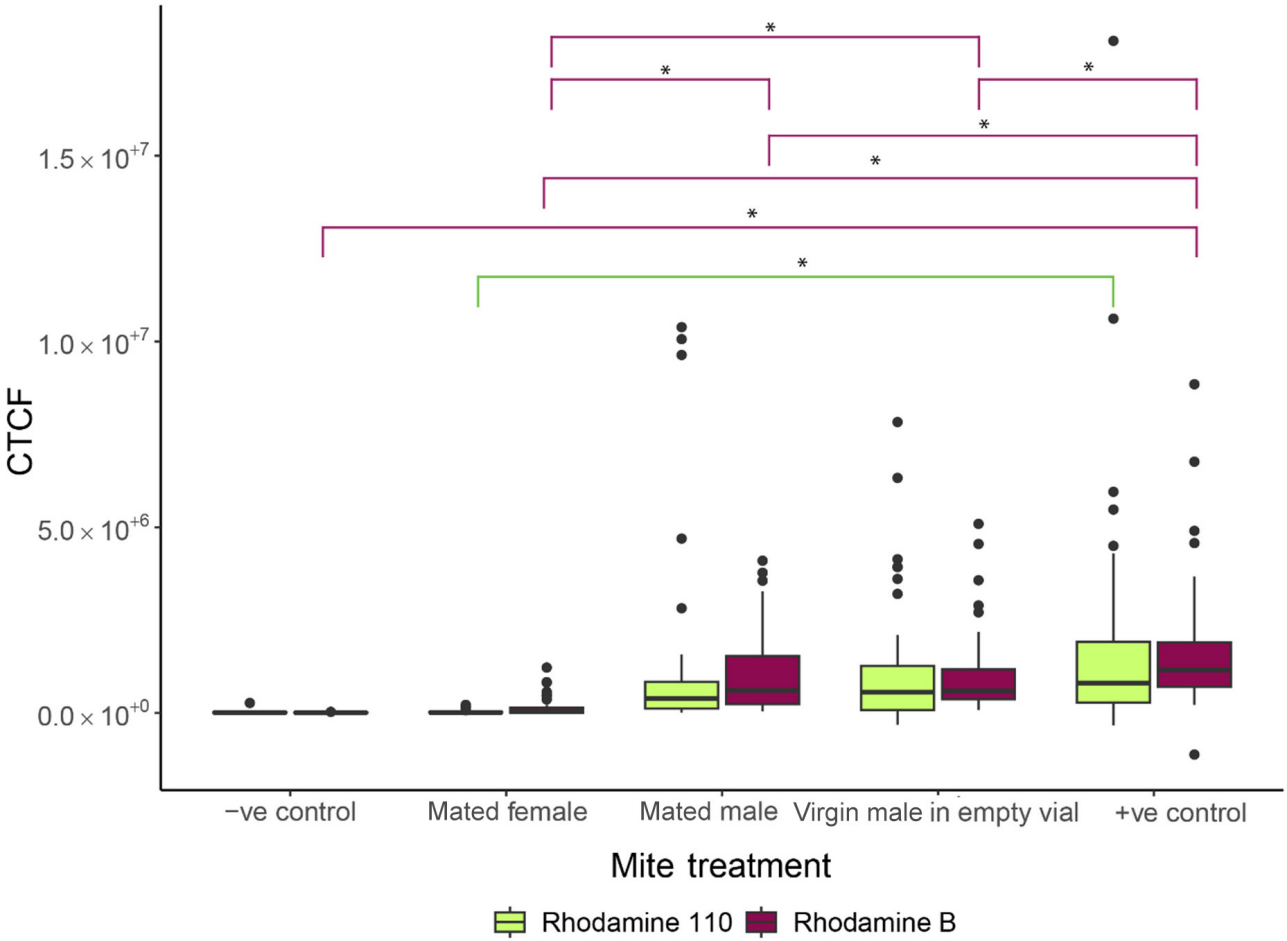
Corrected total cell fluorescence (CTCF) of the sperm transfer treatment for negative control male that was not fed Rhodamine, female mated to a Rhodamine‐treated male, Rhodamine‐treated male mated with a virgin female, Rhodamine‐treated male left in an empty vial, and a positive control male that was kept in a Rhodamine vial in Immu‐mount. Significant differences are outlined using an asterisk and lines matching the colours of the treatment.

Males fed RhB fluoresced less after mating compared with the positive control (i.e., virgin males left in a vial that could continue to feed on Rhodamine) and virgin males left in an empty vial (Figure 4; Table S3). Virgin males left to feed on Rhodamine fluoresced the most compared with all other treatments (Figure 4; Table S3). Additionally, females that mated with RhB‐fed males fluoresced, although the level of fluorescence is not significantly different from the negative control and significantly less than any of the male treatments (Figure 4).

Rh110‐fed males fluoresced similarly after mating compared with virgin males left in a vial that could continue to feed on Rhodamine and virgin males left in an empty vial (Figure 4; Table S3). Additionally, females that mated to males fed Rh110 fluoresce less than males left to feed on Rh110, although the level of fluorescence was not significantly different from the negative control (Figure 4; Table S3). The eggs of females mated to RhB‐ and Rh110‐fed males did not fluoresce (See Figure S3 for reference).

## 4 Discussion

We found that Rhodamine can be used in *Rhizoglyphus echinopus* to tag individuals for behavioural observations. The best concentration to use for the Rhodamine treatment is the same one used by van Reijden, Monchamp, and Lewis (1997) in fireflies. When the mites were fed the original concentration of 4.17 mM of Rhodamine mixed with yeast, we found that we could still identify the individuals with the naked eye and under a regular LED light of a stereo microscope (Figure 1). This visibility allows for observation of individual pre‐copulatory behaviour in more natural social environments. Identifying individuals is even easier under a fluorescent microscope, except that this requires individuals to be euthanised to be mounted, which is more suitable when making morphological measurements after a behavioural trial (Figure 2). Doubling the concentration increased fluorescence in RhB (Figure 2), but in Rh110 the doubling of the concentration lowered the fluorescence in the specimen (Figure 2), most likely because Rh110 was less soluble if too much product is used, and therefore, fewer particles are ingested by the individuals. When mounting the specimen, we found that PBS was not a viable mounting medium as there was too much autofluorescence for RhB and Rh110 treatments. In contrast, the best mounting medium was Immu‐mount, as the fluorescence was less variable for RhB treatments and more visible for Rh110 treatments (Figure 2B,C).

Our trials suggest that there is still some troubleshooting to be done for the use of Rhodamine in post‐copulatory trials of *R. echinopus*. We found that RhB and Rh110 degenerate quickly in the mites unless they are continuously fed more Rhodamine while being left in the Rhodamine vial (Figure 3). The degeneration is even quicker if the male is fed yeast for 24 h after a Rhodamine feeding (Figure 3), which suggests that Rhodamine is likely binding to digestive proteins. Additionally, we found that degeneration occurs when a mite is removed from the Rhodamine treatment and placed in an empty vial for the same amount of time that it took a Rhodamine‐treated male mite to mate, approximately 2 hours (Figure 3). RhB looks more promising for post‐copulatory observations than Rh110, as the degeneration of RhB is slower than that of Rh110, but the differences between mated males and virgin males are too variable (Figure 3). The temperature of our experiments could potentially be lowered from 22°C to decrease the metabolic rate; however, this may not address the issue of individual variability in fluorescence and could alter the behaviour of the mites, making it even more challenging to observe these already slow‐moving animals. As the Rhodamine degeneration in the body of the mite is so quick, we did not run any longevity trials.

We found that mated females fluoresced variably and so dimly that it was impossible to compare ejaculate tailoring of males in females. The light fluorescence that we see is most likely due to some fluorescent ejaculate transferring occurring, but not enough to make conclusive comparisons. The lack of bright fluorescence may be due to the degradation of Rhodamine occurring in the male (Figure 4) and the majority of the Rhodamine binding to the digestive proteins of the male rather than the seminal proteins. As we may be seeing much of the Rhodamine protein binding occurring in the digestive system of the mite, rather than their seminal proteins, there is not enough transfer of Rhodamine to the female to make any conclusive comparisons of ejaculate tailoring or transfer. The rapid degeneration would also explain why the eggs do not fluoresce at all after a female has been mated with a Rhodamine‐treated male. However, the dull fluorescence in the females may also be due to the makeup of the ejaculate of the male. Male mites may not have many ejaculate proteins compared with fruit flies (Sirot et al. 2009) and therefore there are fewer proteins for the Rhodamine to bind to in mite ejaculate. Additionally, the mechanisms underlying sperm production and replenishment in bulb mites remain poorly understood. Further research is required to explore sperm production in bulb mites and whether our approach could be used to explore sperm competition in this species.

We found that Rhodamine can be used to mark individuals of *R. echinopus* and believe that this can be useful in studies of female mate choice, male–male competition and social environmental effects on mating behaviours. *R. echinopus* males are polyphenic, and weapon expression and consequent mating strategies are determined by colony density. Given current research on *R. echinopus*, we have a general picture of male and female mating behaviours in this system, but much information is still missing. Females are likely polyandrous in this system (Radwan 2009), yet we do not know whether they exercise female choice. Fighter males potentially monopolise females by killing rival males (Radwan 2001), but whether this strategy is always exercised by the fighter male may depend on the environmental context—such as the number of females available or the number of rival males. By marking individuals with RhB and Rh110, we can observe mating strategies and morphological differences between morphs in several social contexts, including more natural environments using the naked eye and LED microscopy, where the mites are not euthanised, or through fluorescent microscopy, where they are mounted. In addition, by marking multiple individuals we eliminate the need to constantly observe the animals and remove them from stable environmental conditions as we can check on the vials less frequently. This eliminates the risk of behavioural changes or mating disturbances associated with experimental design. It is necessary to explore whether Rhodamine affects the animals behaviourally through controlled experiments to ensure coloured individuals are not discriminated against in mate‐choice experiments. It would also be important to explore whether Rhodamine affects fecundity or sperm quantity in mites, as this can have negative effects on fitness for both sexes.

Many invertebrate studies are constrained by the number of pairings for mating experiments, resulting in false or unrealistic claims about female choice, male choice, same‐sex competition, and male and female behavioural interactions (Andersson 1994; Andersson and Iwasa 1996). Rhodamine has the potential to be used to mark invertebrates and study them in environments more similar to their natural social environment. In invertebrates, behaviours of interest are prevalent in large social constructs. Model systems such as fruit flies, mites, crickets, beetles and moths are good candidates for the Rhodamine method as these animals are easy to keep in the laboratory and to feed or inject with Rhodamine. They also have translucent abdomens, pupae, eggs or spermatophores, which allow Rhodamine‐tagged individuals to be identified through direct visual observation, without the need of euthanising the individual or observations under a microscope. Using the Rhodamine method to tag these individuals is more practical than fluorescent dusts that can be easily transferred between individuals or cleaned off by the individuals (Still et al. 2014) and is more cost and time effective than GFP (Manier et al. 2010).

While in our system, we found that there is still more troubleshooting to be done with Rhodamine and its use in post‐copulatory experiments, the method might work better with animals of slower metabolism or animals with larger sperm or ejaculate. Although most animals are not as transparent as mites, Rhodamine may still be observed if the animals' genitalia are dissected and mounted, or in the pupae or eggs of the animal. Additionally, because Rhodamine is easily mixed into food, animals that are fed to small invertebrate carnivores such as spiders may be used to explore questions regarding female cryptic choice.

In conclusion, we found that Rhodamine is a cost‐effective and simple way to tag small invertebrates to study pre‐copulatory mating behaviours in a more natural social context, either through direct observation or microscopy of tagged individuals and multi‐choice trials.

## Author Contributions

**Anastasia J. Shavrova:** conceptualization (lead), data curation (lead), formal analysis (lead), investigation (lead), methodology (lead), visualization (lead), writing – original draft (lead). **Bruno A. Buzatto:** conceptualization (supporting), formal analysis (supporting), investigation (supporting), methodology (supporting), resources (lead), supervision (equal), validation (supporting), visualization (supporting), writing – review and editing (equal). **Michael M. Kasumovic:** conceptualization (supporting), data curation (supporting), formal analysis (supporting), funding acquisition (lead), investigation (supporting), methodology (supporting), project administration (supporting), resources (equal), supervision (equal), visualization (supporting), writing – review and editing (equal).

## Conflicts of Interest

The authors declare no conflicts of interest.

## Supporting information


Data S1.

## Data Availability

All data and code are available at here: https://github.com/ajshavrova/Rhodamine_Methods.git.
