# Supplementary material for: Using Rhodamine to Tag Mites for Studies of Pre‐ and Post‐Copulatory Sexual Selection
Source: Ecol Evol. 2024 Nov 5;14(11):e70525. doi: 10.1002/ece3.70525 (PMC11537705; doi:10.1002/ece3.70525)
Supplement: Supplementary file 1 — Data S1. [file ECE3-14-e70525-s001.docx]

# Supplementary Material


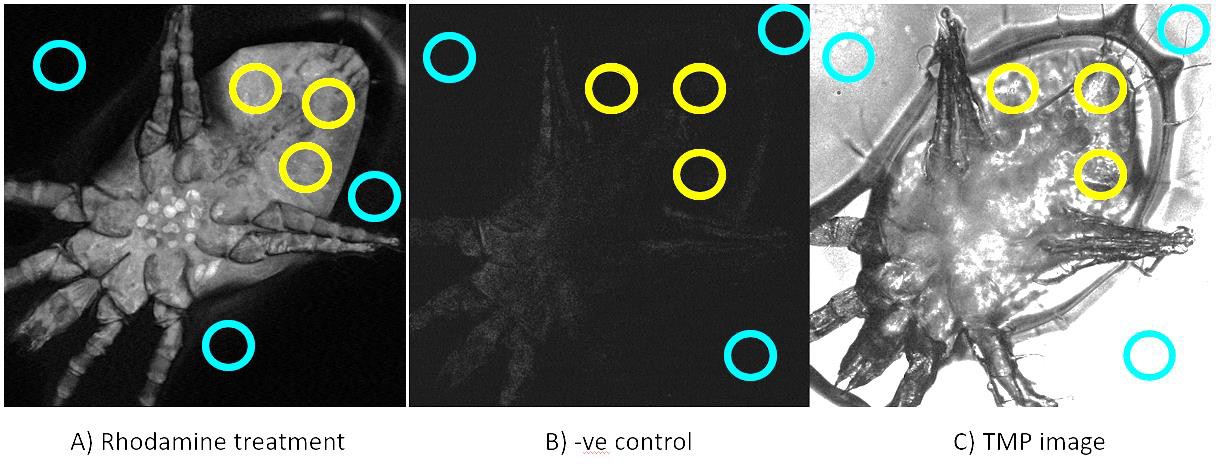


Figure S1. Reference of how the measurements of CTCF were taken using the circle tool in Fiji. Blue circles represent the background measurements and yellow circles represent the body measurements for A) Rhodamine treated fluorescent image, B) negative control image of the negative control for guidance, C) Transmission-photo multiplier (TMP) image for further measurement guidance.

Table S1. Corrected Total Cell Fluorescence (CTCF) estimate contrasts, including standard error (SE) and the degrees of freedom (df), from the linear model comparing two concentrations (original, doubled) and three mounting media (Fluoromount, Immu-mount, Phosphate Buffer Solution (PBS)) for Rhodamine B (RhB) and Rhodamine 110 (Rh110) treatments. Bolded rows are significant.

|  | | Concentration Contrast | Estimate | SE | df | t value | P value |
| --- | --- | --- | --- | --- | --- | --- | --- |
| RhB | Fluoromount | original –  doubled | -234670 | 158203 | 66 | -1.48 | 0.31 |
|  |  | **original – (-ve) control** | **434187** | **170879** | **66** | **2.54** | **0.04** |
|  |  | **doubled – (-ve)**  **control** | **668857** | **170879** | **66** | **3.91** | **0.0006** |
|  | Immu-mount | original –  doubled | -94581 | 158203 | 66 | -0.60 | 0.82 |
|  |  | original – (-ve)  control | 390995 | 170879 | 66 | 2.29 | 0.06 |
|  |  | **doubled – (-ve)**  **control** | **485576** | **170879** | **66** | **2.84** | **0.02** |
|  | PBS | original –  doubled | -19573 | 316405 | 66 | -0.06 | 1.00 |
|  |  | original – (-ve) control | 16316 | 316405 | 66 | 0.05 | 1.00 |
|  |  | doubled – (-ve)  control | 35889 | 316405 | 66 | 0.11 | 1.00 |
| Rh100 | Fluoromount | original –  doubled | 149356 | 108069 | 75 | 1.38 | 0.36 |
|  |  | **original – (-ve) control** | **268776** | **108069** | **75** | **2.49** | **0.04** |
|  |  | doubled – (-ve) control | 119420 | 108069 | 75 | 1.11 | 0.52 |
|  | Immu-mount | **original –**  **doubled** | **486713** | **108069** | **75** | **4.50** | **0.0001** |
|  |  | **original – (-ve) control** | **622771** | **108069** | **75** | **5.76** | **<0.0001** |
|  |  | doubled – (-ve) control | 136058 | 108069 | 75 | 1.26 | 0.42 |
|  | PBS | original –  doubled | 208408 | 216138 | 75 | 0.96 | 0.60 |
|  |  | original – (-ve) control | 249571 | 187181 | 75 | 1.33 | 0.38 |
|  |  | doubled – (-ve) control | 41163 | 187181 | 75 | 0.22 | 0.97 |

Table S2. Corrected Total Cell Fluorescence (CTCF) estimate contrasts, including the standard error (SE) and the degrees of freedom (df), from the linear model comparing different degradation treatments (empty vial, yeast fed, positive control, negative control) and mounting media (Fluoromount, Immu-mount) for Rhodamine B (RhB) and Rhodamine 110 (Rh110). Bolded rows are significant.

|  | | Treatment Contrast | Estimate | SE | df | t value | P value |
| --- | --- | --- | --- | --- | --- | --- | --- |
| RhB | Fluoromount | **Empty vial – (+ve) control** | **-451670** | **97090** | **115** | **-4.65** | **0.0001** |
|  |  | **Fed yeast – (+ve)**  **control** | **-523393** | **93043** | **115** | **-5.63** | **<0.0001** |
|  |  | Empty vial – (+ve) control | 99853 | 127121 | 115 | 0.79 | 0.86 |
|  |  | Fed yeast – (-ve) control | 28129 | 124057 | 115 | 0.23 | 1.00 |
|  |  | Empty vial – fed yeast | 71723 | 100018 | 115 | 0.72 | 0.89 |
|  |  | **(+ve) control –**  **(-ve) control** | **-551522** | **121709** | **115** | **-4.53** | **0.0001** |
|  | Immu-mount | Empty vial – (+ve) control | -279536 | 121709 | 115 | -2.30 | 0.10 |
|  |  | **Fed yeast – (+ve) control** | **-377145** | **121709** | **115** | **-3.10** | **0.01** |
|  |  | Empty vial – (-ve) control | 158750 | 146786 | 115 | 1.08 | 0.70 |
|  |  | Fed yeast – (-ve) control | 61141 | 146786 | 115 | 0.42 | 0.98 |
|  |  | Empty vial – fed yeast | 97609 | 146786 | 115 | 0.67 | 0.91 |
|  |  | **(+ve) control –**  **(-ve) control** | **-438285** | **121709** | **115** | **-3.60** | **0.0026** |
| Rh110 | Fluoromount | Empty vial – (+ve) control | -124360 | 62596 | 145 | -1.99 | 0.20 |
|  |  | Fed yeast – (+ve) control | -156132 | 66672 | 145 | -2.34 | 0.09 |
|  |  | Empty vial – (-ve) control | 69738 | 77412 | 145 | 0.901 | 0.80 |
|  |  | Yeast fed – (-ve) control | 37966 | 80743 | 145 | 0.47 | 0.97 |
|  |  | Empty vial – fed yeast | 31772 | 64920 | 145 | 0.49 | 0.96 |
|  |  | (+ve) control –  (-ve) control | -194098 | 78887 | 145 | -2.46 | 0.07 |
|  | Immu-mount | **Empty vial – (+ve) control** | **-298380** | **69572** | **145** | **-4.29** | **0.0002** |
|  |  | **Fed yeast – (+ve) control** | **-295358** | **73440** | **145** | **-4.02** | **0.0005** |
|  |  | Empty vial – (-ve) control | 81034 | 8315 | 145 | 0.98 | 0.76 |
|  |  | Fed yeast – (-ve) control | 84056 | 86416 | 145 | 0.97 | 0.77 |
|  |  | Empty vial – fed yeast | -3022 | 78006 | 145 | -0.04 | 1.00 |
|  |  | **(+ve) control –**  **(-ve) control** | **-379414** | **78887** | **145** | **-4.810** | **<0.0001** |

Table S3. Corrected Total Cell Fluorescence (CTCF) estimate contrasts, including the standard error (SE) and the degrees of freedom (df), from the linear model comparing between different sperm transfer treatments (negative control, mated female, mated male, virgin male left in empty vial, positive control) all mounted in Immu-mount for Rhodamine B (RhB) and Rhodamine 110 (Rh110) fed individuals. Bolded rows are significant.

| Treatment Contrast | | Estimate | SE | df | t value | p value |
| --- | --- | --- | --- | --- | --- | --- |
| **RhB** | Mated female – (-ve) control | -135157 | 427884 | 172 | -0.316 | 1.00 |
|  | Mated male – (-ve) control | -1049530 | 427884 | 172 | -2.453 | 0.11 |
|  | Virgin male – (-ve) control | -1019221 | 423668 | 172 | -2.41 | 0.12 |
|  | **Pos. control** – **(-ve)** **control** | **-1856277** | **451029** | **172** | **-4.12** | **0.0006** |
|  | **Mated female –**  **mated male** | **-914373** | **247039** | **172** | **-3.70** | **0.003** |
|  | **Mated female –**  **virgin male** | **-884064** | **239663** | **172** | **-3.69** | **0.003** |
|  | **Mated female –**  **(+ve) control** | **-1721119** | **285256** | **172** | **-6.03** | **<0.0001** |
|  | Mated male – virgin male | 30309 | 239663 | 172 | 0.126 | 1.00 |
|  | **Mated male – (+ve) control** | **-806746** | **285256** | **172** | **-2.83** | **0.04** |
|  | **Virgin male– (+ve) control** | **-837056** | **278893** | **172** | **-3.00** | **0.03** |
| **Rh110** | Mated female – (-ve) control | 211 | 907074 | 166 | 0.00 | 1.00 |
|  | Mated male – (-ve) control | -1160637 | 827006 | 166 | -1.40 | 0.63 |
|  | Virgin male– (-ve) control | -041100 | 816632 | 166 | -1.28 | 0.71 |
|  | Pos. control – (-ve) control | -2032961 | 848490 | 166 | -2.40 | 0.12 |
|  | Mated female –  mated male | -1160849 | 595672 | 166 | -1.95 | 0.30 |
|  | Mated female – virgin male | -1041312 | 581183 | 166 | -1.79 | 0.38 |
|  | **Mated female –**  **(+ve) control** | **-2033173** | **625157** | **166** | **-3.25** | **0.01** |
|  | Mated male – virgin male | 119537 | 446015 | 166 | 0.268 | 1.00 |
|  | Mated male – (+ve) control | -872324 | 501973 | 166 | -1.74 | 0.41 |
|  | Virgin male – (+ve) control | -991861 | 484692 | 166 | -2.05 | 0.25 |


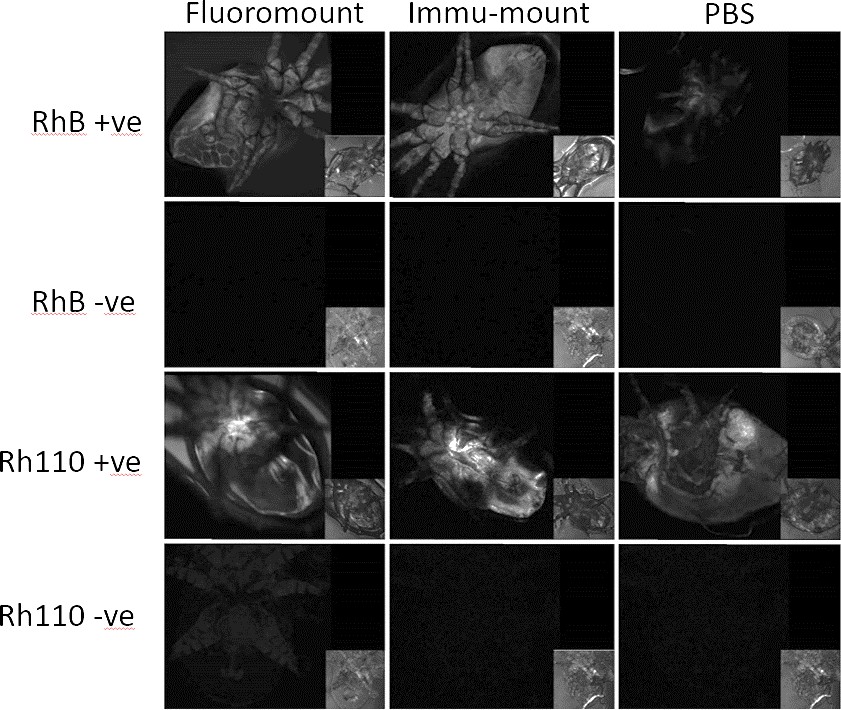


Figure S2. Zeiss LSM 880 images of Rhodamine treated mites and the negative controls, with all corresponding TMP images, in three different mounting media (Fluoromount, Immu-mount, PBS).


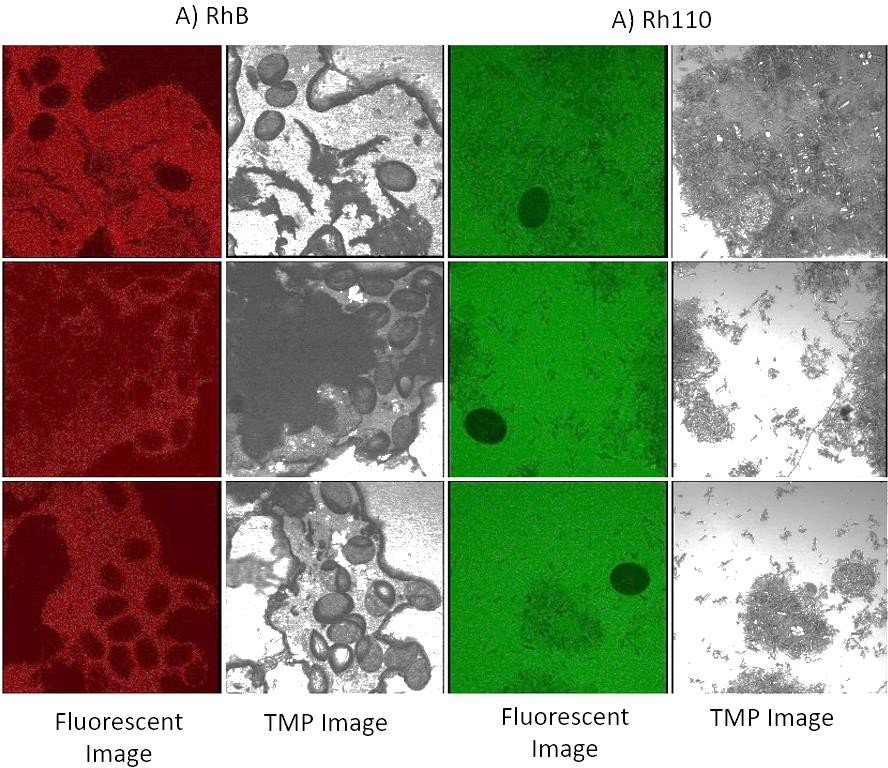


Figure S3. Zeiss LSM 880 images of eggs laid by females mated with A) RhB treated males and B) Rh110 treated males. The left image is the fluorescent image with corresponding wavelength for each Rhodamine type and the right image is the TMP image for reference.

Equation S1. Male fitness calculation in a social context treatment

𝑇𝑜𝑡𝑎𝑙 𝑒𝑔𝑔 𝑐𝑜𝑢𝑛𝑡 𝑓𝑟𝑜𝑚 𝑜𝑛𝑒 𝑓𝑒𝑚𝑎𝑙𝑒

𝑀𝑎𝑙𝑒 𝐹𝑖𝑡𝑛𝑒𝑠𝑠 =

(𝑀𝑎𝑙𝑒𝑠 𝑡ℎ𝑎𝑡 𝑚𝑜𝑢𝑛𝑡𝑒𝑑 𝑓𝑒𝑚𝑎*le)*
